# The Common Partner of Several Methyltransferases TRMT112 Regulates the Expression of N6AMT1 Isoforms in Mammalian Cells

**DOI:** 10.3390/biom9090422

**Published:** 2019-08-28

**Authors:** Lilian Leetsi, Kadri Õunap, Aare Abroi, Reet Kurg

**Affiliations:** Institute of Technology, University of Tartu, 50411 Tartu, Estonia

**Keywords:** methyltransferase, TRMT112, alternative splicing, N6AMT1, protein stability

## Abstract

Methylation is a widespread modification occurring in DNA, RNA and proteins. The N6AMT1 (HEMK2) protein has DNA N6-methyladenine as well as the protein glutamine and histone lysine methyltransferase activities. The human genome encodes two different isoforms of N6AMT1, the major isoform and the alternatively spliced isoform, where the substrate binding motif is missing. Several RNA methyltransferases involved in ribosome biogenesis, tRNA methylation and translation interact with the common partner, the TRMT112 protein. In this study, we show that TRMT112 regulates the expression of N6AMT1 isoforms in mammalian cells. Both isoforms are equally expressed on mRNA level, but only isoform 1 is detected on the protein level in human cells. We show that the alternatively spliced isoform is not able to interact with TRMT112 and when translated, is rapidly degraded from the cells. This suggests that TRMT112 is involved in cellular quality control ensuring that N6AMT1 isoform with missing substrate binding domain is eliminated from the cells. The down-regulation of TRMT112 does not affect the N6AMT1 protein levels in cells, suggesting that the two proteins of TRMT112 network, WBSCR22 and N6AMT1, are differently regulated by their common cofactor.

## 1. Introduction

Methylation is an essential epigenetic modification that occurs on a wide variety of substrates. Human genome encodes more than 200 methyltransferases and the majority of them binds S-adenosylmethionine (AdoMet) and uses it as a methyl donor in methyl transfer reactions [1,2]. About 30% of human methyltransferases have been associated with disorders, most frequently with cancers and mental disorders [1]. Several epigenetic drugs have been approved or are currently in clinical trials for cancer therapy, including inhibitors of DNA methyltransferases, histone methyltransferases and lysine specific demethylases [3,4].

Many methyltransferases require accessory proteins for their activity and stability [5,6]. TRMT112 is a small evolutionarily conserved protein that acts as a cofactor for different methyltransferases involved in rRNA, tRNA, DNA and protein methylation [7,8,9,10]. First, TRMT112 interacts with 18S rRNA methyltransferase WBSCR22 (also known as BUD23, MERM1 and RNMT2), which is involved in pre-rRNA processing and ribosome 40S subunit biogenesis [8,11,12]. The WBSCR22 protein, but not its methyltransferase activity, is required for ribosome biogenesis in humans [8]. It has also been shown that WBSCR22 modulates the histone methylation of GILZ and Zac1 promoter, although the histone methylation activity has not been shown in vitro [13,14]. Second, the TRMT112 protein interacts with tRNA-hypermodification enzyme ALKBH8, which is required for DNA damage survival [15,16]. In addition to the methyltransferase domain, which catalyzes the methylation of cm^5^U to mcm^5^U in several tRNAs, ALKBH8 has also an oxygenase domain, which catalyzes hydroxylation of wobble 5-methoxycarbonylmethyluridine, whereas both enzymatic activities can work independently [17]. The third protein that interacts with TRMT112 is TRMT11 [6]. Yeast orthologous complex Trm11-Trm112 methylates the guanosine at position 10 of some tRNAs that stabilizes the tRNA structure [18,19].

The fourth TRMT112 interactor, the methyltransferase N6AMT1 (HEMK2) protein has DNA N6-methyladenine as well as the protein methyltransferase activity [10,20,21]. N6AMT1 has a GxGxG domain essential for AdoMet binding and a substrate binding domain NPPY that is distinctive to DNA methyltransferases, however, it was first shown to have a protein methyltransferase activity catalyzing the N5-methylation of the glutamine residue in the GGQ motif of eukaryotic translation termination factor 1 (ETF1) [10], and proteins involved in chromatin remodeling, including CHD5 (chromodomain helicase DNA-binding protein 5) and NUT (nuclear protein in testis) [22]. For protein methylation activity, N6AMT1 requires the binding partner TRMT112, N6AMT1 alone is not sufficient to methylate the proteins [10,20]. Structural studies in *Saccharomyces cerevisiae* have shown that the yeast Mtq2-Trm112 interaction triggers a conformational change of Mtq2, which strongly increases the binding of AdoMet [23]. The N6AMT1-TRMT112 heterodimer, also called “lysine methyltransferase 9” (KMT9), possesses the histone lysine methyltransferase activity writing the monomethylated chromatin mark on H4K12 in vitro and in vivo. Methylation of H4 by N6AMT1 controls the expression of genes encoding proteins involved in the cell cycle, as well as androgen receptor-dependent and castration-resistant proliferation of prostate tumor cells [20]. N6AMT1 protein can also methylate monomethylarsonous acid (3+) into the less toxic dimethylarsonic acid, thus playing a role in the modulation of arsenic-induced toxicity, although it only has a minor role compared to arsenic (+3 oxidation state) methyltransferase AS3MT [24].

We have previously shown that TRMT112 is the main interaction partner for the WBSCR22 protein. The stability of WBSCR22 protein is regulated by the interaction with the TRMT112 protein and the level of WBSCR22 protein in the cells is controlled by the 26S proteasome pathway. In this work, we examined the interaction between TRMT112 and N6AMT1 proteins. We identified two isoforms of N6AMT1, both expressed in equal quantities on mRNA level, but only the main isoform was detected on the protein level. We show that the alternatively spliced isoform of N6AMT1, formed through exon skipping, does not form a complex with TRMT112 protein. Point mutations near the interaction surface of N6AMT1 disrupt the interaction with TRMT112 protein, suggesting that amino acids missing in alternatively spliced isoform are required for the formation of TRMT112-N6AMT1 heterodimer. The down-regulation of TRMT112 does not affect the N6AMT1 protein levels in cells, thus the two proteins of TRMT112 network, WBSCR22 and N6AMT1, are differently regulated by their common co-factor.

## 2. Materials and Methods

### 2.1. Plasmids

The coding region of N6AMT1 was amplified from uman osteosarcoma cells (U2OS) cells by PCR using primers N6AMT1_BamHI_F (5′-CGCGGATCCACTATGGCAGGGGAGAAC-3′) and N6AMT1_KpnI_R (5′-GGTACCTGCATTCAGTTTCCAGTAG-3′). The amplification resulted in two products of different sizes, which corresponded to two N6AMT1 isoforms by sequencing. Both products were cloned into pJET1.2/blunt vector (Thermo Scientific, Waltham, MA, USA). pJET1.2-N6AMT1-iso1 and pJET1.2-N6AMT1-iso2 were cut with BamHI, KpnI and the inserts were cloned into BamHI and KpnI sites of pQM-CMV-N/A (Icosagen, Tartu, Estonia), yielding pQM-N6AMT1-iso1 and pQM-N6AMT1-iso2. For EGFP expression vectors, coding sequences of both N6AMT1 isoforms were cut from pJET1.2 vectors with BamHI, KpnI and cloned into BglII, KpnI sites of pEGFP-C1 (Colontech, Mountain View, CA, USA), yielding pEGFP-N6AMT1-iso1 and pEGFP-N6AMT1-iso2. Point mutations R111D117/AA were generated by PCR and the sequence was cut from pUC57-N6AMT1-R111D117/AA with EcoRV, BglII and cloned into EcoRV and BglII sites of pEGFP-N6AMT1-iso1, yielding pEGFP-N6AMT1-R111D117/AA. WBSCR22 expression plasmids pQM-NTag-WBSCR22 [11] and pEGFP-WBSCR22 [9], used in this study as a positive control, have been described previously.

### 2.2. Cell Culture and Transfections

Human osteosarcoma cells (U2OS) and human cervical carcinoma cells (HeLa), obtained from ATCC (American Type Culture Collection; Manassas, VA, USA), were grown in Iscove’s Modified Dulbecco’s Media (IMDM) supplemented with 10% fetal calf serum (FCS), 100 U/mL penicillin and 100 µg/mL streptomycin. Cells were incubated at 37 °C in 5% CO2 environment. For protein expression analysis, 250 µL of U2OS cells (2 × 10^6^) were mixed with salmon sperm carrier DNA and 3 µg of pEGFP-WBSCR22, pEGFP-N6AMT1iso1 or pEGFP-N6AMT1iso2 plasmids and transfected by electroporation in 4 mm cuvettes (Thermo Fisher Scientific) using Bio-Rad (Hercules, CA, USA) GenePulser Xcell (settings 200 V, 975 µF). The cells were suspended in IMDM medium supplemented with 10% FCS and antibiotics on 100 mm cell culture dishes and analyzed 24 h after transfection by Western blot. Alternatively, U2OS cells were transfected with 1 µg of pQM-CMV-E2-N/A (Icosagen), pQM-WBSCR22, pQM-N6AMT1iso1 and pQM-N6AMT1iso2. Each transfection was aliquoted onto two 60 mm cell culture plates. 24 h later cells were treated with proteasome inhibitor MG132 and analyzed by Western blot. To study the interaction with TRMT112 protein, U2OS cells were transfected with 3 µg of pEGFP-C1, pEGFP-WBSCR22, pEGFP-N6AMT1iso1, pEGFP-N6AMTiso2 and incubated in cell culture media for 24 h before adding MG132 and analyzing by co-immunoprecipitation. HeLa cells were transfected with 3 µg of pEGFP-C1, pEGFP-WBSCR22, pEGFP-N6AMT1iso1, pEGFP-N6AMT1-R111D117/AA and pEGFP-N6AMTiso2. The transfections were aliquoted onto two 100 mm cell culture dishes. 24 h post transfection MG132 was added and the cells were later analyzed by co-immunoprecipitation.

### 2.3. RNA Interference

Interfering RNAs siNeg and siTRMT112 #4 and #1 used in this study have been described previously [9]. For siRNA knock-down, Lipofectamine RNAiMAX (Thermo Fisher Scientific) was used following manufacturers reverse transfection protocol. HeLa cells were transfected with 250 pmol of control and TRMT112 siRNAs using 12.5 µL lipofectamine RNAiMAX on 60 mm cell culture dishes. For U2OS cells, 250 pmol siRNAs were used for transfection on 100 mm cell culture dishes. HeLa and U2OS cells were analyzed 72 h after transfection by Western blot.

### 2.4. Treatment with MG132

Twenty-four hours post transfection with pQM-CMV-E2-N/A (Icosagen), pQM-WBSCR22, pQM-N6AMT1iso1 and pQM-N6AMT1iso2, the U2OS cells were treated with proteasome inhibitor MG132 (Merck KGaA, Darmstadt, Germany) at a concentration of 0 µM and 5 µM for 16 h and analyzed by western blot. For immunoprecipitation analysis, U2OS cells were transfected with pEGFP-C1 (Takara Bio Group; Kusatsu, Shiga, Japan), pEGFP-WBSCR22, pEGFP-N6AMT1iso1, pEGFP-N6AMTiso2 and after 24 h treated with MG132 for 16 h. HeLa cells transfected with pEGFP-C1, pEGFP-WBSCR22, pEGFP-N6AMT1iso1, pEGFP-N6AMTiso2 and pEGFP-N6AMT1-R111D117/AA were treated with MG132 for 16 h and analyzed by immunoprecipitation assay.

### 2.5. Immunoprecipitation and Immunoblotting

U2OS cells transfected with 3 µg of plasmids pEGFP-C1, pEGFP-WBSCR22, pEGFP-N6AMT1iso1, pEGFP-N6AMTiso2 and treated with 5 µM MG132 were used for co-immunoprecipitation with GFP-Trap_M magnetic beads (ChromoTek GmbH, Munich, Germany) following the manufacturer’s protocol. Alternatively, co-immunoprecipitation was carried out with HeLa cells transfected with 3 µg of pEGFP-C1, pEGFP-WBSCR22, pEGFP-N6AMT1iso1, pEGFP-N6AMT1-R111D117/AA, pEGFP-N6AMTiso2 and treated with 0 µM or 5 µM MG132.

The proteins were detected by western blot using the mouse monoclonal antibodies anti-E2-Tag antibody 5E11 (Icosagen), anti-α-tubulin (Sigma-Aldrich, St. Louis, MO, USA) and rabbit polyclonal antibodies against TRMT112 (HPA04006, Sigma-Aldrich), WBSCR22 (sc-135322; Santa Cruz Biotechnology, Dallas, TX, USA), EGFP (Institute of Technology; University of Tartu, Tartu, Estonia) and N6AMT1 (ab173804; Abcam, Cambridge, UK). Detection was performed using an ECL detection kit (GE Healthcare, Chicago, IL, USA) following the manufacturer’s instructions.

### 2.6. Protein Alignment and Interaction Modeling on Protein Structures

Human N6AMT1 iso1/iso2 and yeast Mtq2 sequence alignment was created based on blastp search results (https://blast.ncbi.nlm.nih.gov/Blast.cgi). Human TRMT112 and human N6AMT1 sequences (TR112_HUMAN and N6MT1_HUMAN respectively) were downloaded from Uniprot database (uniprot.org) and modeled with Modeller Software (Modeller version 9.21; downloaded from salilab.org/modeller/; developed by the University of California, San Francisco, CA, USA; and Accelrys, San Diego, CA, USA;) using the structure of the *Encephalitozoon cuniculi* Mtq2-Trm112 complex (PDB: 3Q87) as a template. The modeled structure was visualized with Jmol (http://www.jmol.org/).

## 3. Results

### 3.1. The Stability of N6AMT1 Isoforms Is Regulated by Proteasomes

**Figure 1:**
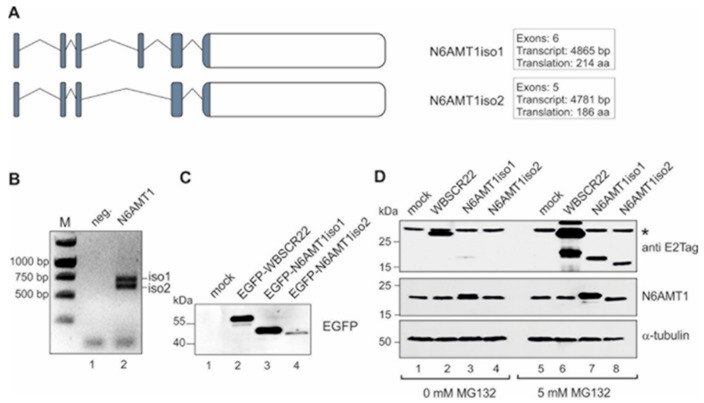
The human genome encodes two different isoforms of N6AMT1. (**A**) A schematic representation of N6AMT1 two isoforms. The coding sequence is shaded gray. (**B**) The expression of N6AMT1iso1 and N6AMT1iso2 on the mRNA level was detected by PCR from the cDNA of human osteosarcoma cells (U2OS) cells. (**C**) Protein expression of N6AMT1iso and N6AMT1iso2. U2OS cells were transfected with plasmids encoding EGFP-N6AMT1iso1 and EGFP-N6AMT1iso2. Cells were harvested 24 h after transfection and analyzed by western blot using an anti-EGFP antibody. (**D**) The stability of N6AMT1 isoforms is regulated by ubiquitin-proteasome pathway. U2OS cells were transfected with plasmids encoding WBSCR22, N6AMT1iso1 and N6AMT1iso2 and 24 h later treated with 0 µM or 5 µM MG132 for 16 h and analyzed by immunoblotting. Western blot analysis was performed with antibodies against E2Tag (recombinant protein), N6AMT1 (endogenous as well as recombinant protein) and α-tubulin (cellular control). The unspecific band detected with anti-E2Tag is shown by an asterisk.

The human genome encodes two alternatively spliced isoforms of N6AMT1, isoform 1 and isoform 2 (ensembl.org; Figure 1A). The main isoform N6AMT1iso1 contains six exons, the alternatively spliced isoform 2 has five exons, as exon 4 encoding for the substrate binding motif NPPY is missing [25]. In order to clone the N6AMT1 coding sequence into the expression vector, we amplified the coding region of N6AMT1 by PCR from the cDNA of U2OS cells and got two products with different sizes, but in equal quantities (Figure 1B), corresponding to N6AMT1 isoforms according to the Ensemble database. Both isoforms were verified by sequencing and found to correspond to respective sequences of the database. We cloned both isoforms of N6AMT1 into the mammalian expression vector, in-frame with EGFP protein and E2Tag epitope tag, under the control of the strong CMV promoter similar to RNA methyltransferase WBSCR22 [9]. The expression vectors were transfected into U2OS cells and protein expression was analyzed by western blotting. As shown in Figure 1C, the expression of EGFP-N6AMT1iso1 was readily detected and comparable to EGFP-WBSCR22 fusion protein, while EGFP-N6AMT1iso2 was detected at a very low level. Performing the immunoblot analysis of N6AMT1iso1 and N6AMT1iso2 without N-terminal EFGP protein, we could detect only isoform 1, though at a much lower level than WBSCR22, but not N6AMT1iso2 (Figure 1D; lanes 3 and 4). The mock control analyzed with N6AMT1 specific antibody exhibited one band suggesting that U2OS cells express only one isoform of N6AMT1 protein, the isoform 1 (Figure 1D, lane 1) as a stable protein.

We have previously shown that the expression of WBSCR22 protein is regulated by an ubiquitin-proteasome pathway [9]. To analyze whether the N6AMT1 protein expression is also regulated by proteasomes, we treated the transfected cells with proteasome inhibitor MG132. MG132 was added to a final concentration of 5 µM 24 h after transfection and the cells were analyzed 16 h later by immunoblotting. As shown on Figure 1D, the protein level of all three proteins increased in the presence of MG132 (upper panel; lanes 6–8) comparing to the non-treated cells (lanes 2–4). The inhibition of proteasomes increased the protein level of both N6AMT1 isoforms, but did not affect the level of α-tubulin, which refers that the level of N6AMT1 proteins is controlled by proteasomes. Again, only one isoform, N6AMT1iso1 was detected in mock control (Figure 1, central panel; lane 5), in U2OS cells, under these conditions.

### 3.2. N6AMT1 iso2 Does Not Interact with TRMT112

**Figure 2:**
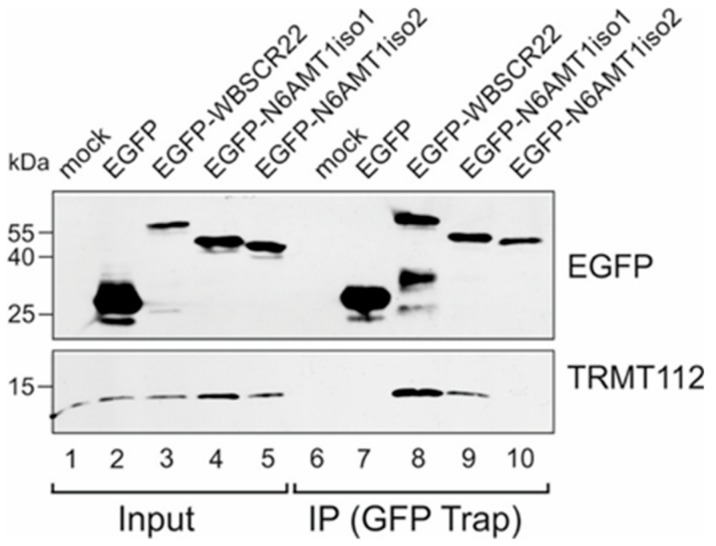
N6AMT1iso2 does not interact with TRMT112. U2OS cells were transfected with plasmids encoding EGFP, EGFP-WBSCR22, EGFP-N6AMT1iso1, EGFP-N6AMT1iso2 and 24 h later treated with 5 µM MG132 for 16 h and analyzed by co-immunoprecipitation with magnetic beads covalently coupled with the EGFP binding protein. Immunoblotting was carried out with antibodies against EGFP and TRMT112.

The interaction of N6AMT1/HEMK2 protein and the yeast orthologue Mtq2 with the TRMT112 protein has been shown previously [10,23]. However, it is not known, whether the shorter isoform of N6AMT1 is also able to form a complex with TRMT112. To analyze the ability of N6AMT1iso2 to interact with TRMT112, we performed the co-immunoprecipitation assay with EGFP-tagged N6AMT1iso1 and N6AMT1iso2 using the GFP-Trap system (ChromoTek). Protein complexes were pulled down from U2OS cells transfected with expression plasmids, and treated with proteasome inhibitor MG132 to ensure the expression of both isoforms of N6AMT1, and analyzed by immunoblotting with anti-EGFP and TRMT112 antibodies. We have previously shown that WBSCR22 protein co-immunoprecipitates with TRMT112 [9] and this was used as a positive control. As shown in Figure 2, both WBSCR22 and N6AMT1iso1 pulled down the endogenous TRMT112 protein (lanes 8 and 9), whereas N6AMT1iso2 protein did not (lane 10). These data show that N6AMT1iso2, which lacks 28 amino acids in the middle of the protein, is not able to interact with TRMT112.

**Figure 3:**
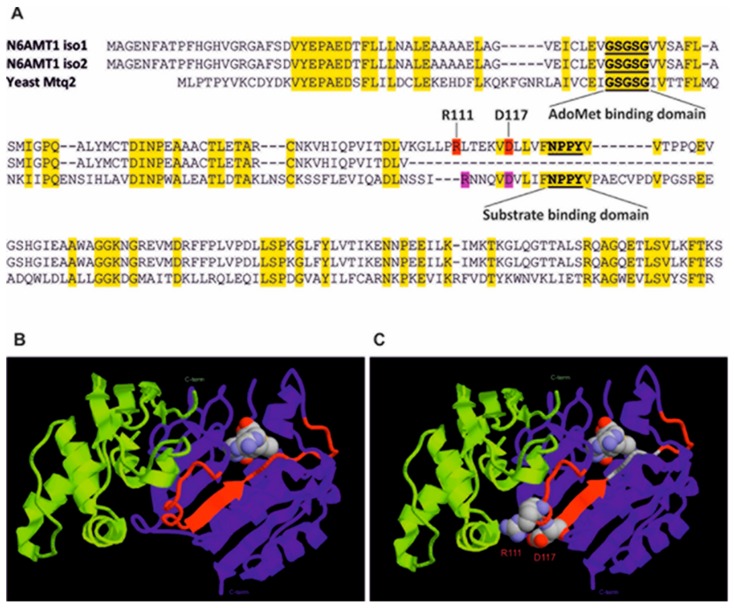
Model of the human TRMT112 and N6AMT1 protein complex. (**A**) Alignment of N6AMT1 isoforms with yeast Mtq2 sequence was created based on blastp results. Yellow highlights mark identical amino acids shared between human N6AMT1 and yeast Mtq2 proteins. Two highly conserved amino acids (human R111 and D117) on both TRMT112-binding methyltransferases are highlighted with red. Substrate binding motif NPPY and AdoMet binding motif GxGxG are underlined and marked in bold. (**B**) Structural model of N6AMT1 and TRMT112 interaction, that is modeled with a Modeler using the *Encephalitozoon cuniculi* Mtq2-Trm112 complex (PDB code 3Q87) as a template. Twenty-eight amino acids present in isoform 1, but missing in isoform 2 are colored red. AdoMet is marked with spacefill in ribbon structure. (**C**) The two conserved amino acids R111 and D117 are highlighted as balls in ribbon structure.

Next we created a sequence alignment of human N6AMT1 iso1/iso2 and yeast Mtq2 based on a blastp search results (https://blast.ncbi.nlm.nih.gov/Blast.cgi; Figure 3A). Identical residues shared by both human N6AMT1 and yeast Mtq2 are marked yellow. The crystal structure of yeast Mtq2-Trm112 complex bound to its co-factor SAM has been resolved [23] and based on this, we created a model of human N6AMT1-TRMT112 protein complex (Figure 3B) by using a corresponding Mtq2-Trm112 crystal structure from *E. cuniculi* as a template. In Figure 3B, the 28 amino acids present in isoform 1, but missing in isoform 2 are colored red.

The eukaryotic Trm112 is a partner for at least four methyltransferases from which three complexes, *Ecu*Mtq2-Trm112 [23], *Sc*Bud23-Trm112 [26] and *Yl*Trm9-Trm112 [27] have been crystallized. These structures revealed that Trm112 interacts in a very similar way with its partners [6]. In all cases, a ß-zipper interaction formed between the ß-strands from both proteins resulted in a continuous large eleven-stranded ß-sheet, generating the hydrophobic interface to the Trm112-MTase complex. In addition, the structure based alignment between yeast Trm9, Bud23, Trm11 and Mtq2 showed “conserved hotspots” involved in complex formation [6]. These amino acids are E101, R112 and D117 of yeast Mtq2 and are marked red in Figure 3A. Two of them, R111 (corresponding to R112 in Mtq2) and D117 are missing in isoform 2 of N6AMT1 (Figure 3A). The two conserved amino acids R111 and D117 are highlighted in Figure 3B as balls in ribbon structure. In our model, R111 and D117 locate in close proximity to TRMT112 protein surface arising the possibility that they may be involved in a N6AMT1-TRMT112 complex formation similar to the yeast Mtq2-Trm112 complex. Recently the crystal structure of the human N6AMT1-TRMT112 complex together with SAH was published [20] and this allowed us to compare our model with the actual crystal structure. As shown in Figure S1A, the backbone was very similar, but there were slight differences in positions of some loops. One of the differences was the orientation of the Arg111 side chain, which was oriented more towards the TRMT112 surface than initially expected, raising the possibility that it is important for the formation of the N6AMT1-TRMT112 complex (Supplementary Figure S1A).

**Figure 4:**
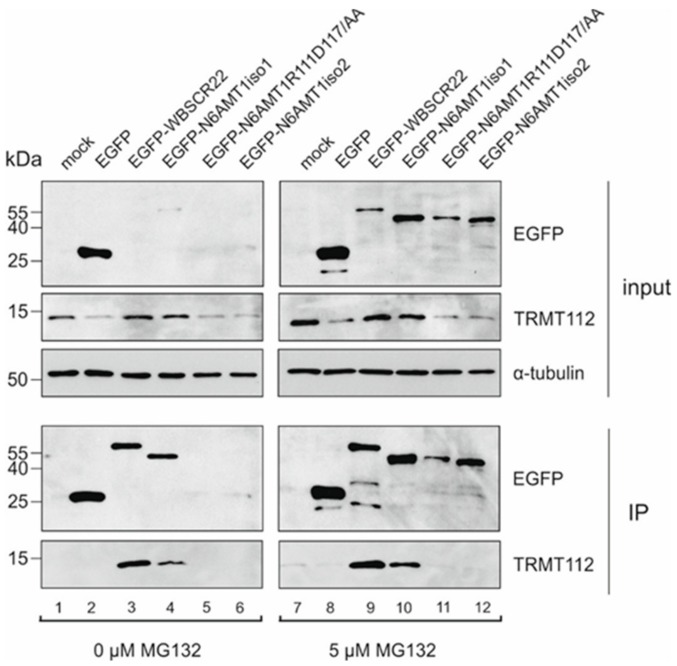
The N6AMT1 mutant does not bind to TRMT112. Human cervical carcinoma cells (HeLa) cells were transfected with plasmids encoding EGFP, EGFP-WBSCR22, EGFP-N6AMT1iso1, EGFP-N6AMT1-R111D117/AA, EGFP-N6AMT1iso2 and 24 h later treated with 0 µM or 5 µM MG132 for 16 h and analyzed by co-immunoprecipitation with magnetic beads covalently coupled with the EGFP binding protein. Immunoblotting was carried out with antibodies against EGFP, TRMT112 and α-tubulin. Input represents 1/10 of material.

In order to test the role of R111 and D117 of N6AMT1 in complex formation with TRMT112, we constructed a double-mutant N6AMT1-R111D117/AA, where Arg111 and Asp117 were replaced with alanines. Then EGFP-N6AMT1iso1, N6AMT1-R111D117/AA and EGFP-N6AMT1iso2 together with EGFP, EGFP-WBSCR22 as controls were expressed in cells with and without the presence of proteasome inhibitor MG132 and immunoprecipitation using the GFP-Trap system was performed. As shown in Figure 4, we were able to pull-down TRM112 with both N6AMT1iso1 and WBSCR22 in spite of their very low levels in input material without MG132 (lanes 3 and 4). The proteasome inhibitor was added to ensure the expression of N6AMT1iso2 and mutant N6AMT1-R111D117/AA for immunoprecipitation assay (Figure 4; lanes 11 and 12). In spite of this, the mutant protein N6AMT1-R111D117/AA was less stable than wild-type N6AMT1 (Figure 4; compare lanes 10 and 11). In the presence of the proteasome inhibitor, again, WBSCR22 and the major isoform of N6AMT1, N6AMT1iso1, pulled down the TRMT112 protein (lanes 9 and 10), but N6AMT1iso2 was not able to do this (lane 12). The mutant protein, N6AMT1-R111D117/AA showed a similar phenotype to the isoform 2 (lanes 11 and 12), both were defective in forming a complex with the TRMT112 protein. We also noticed that both N6AMT1iso2 and mutant N6AMT1-R111D117/AA were less stable than the full-length protein. Furthermore, expression of recombinant WBSCR22 and N6AMT1 enhanced also the expression level of endogenous TRMT112 in the cells, which was not observed in case of N6AMT1iso2 and mutant N6AMT1-R111D117/AA proteins (Figure 4; input panel; TRMT112 images).

Next we calculated the energetic effect of alanine mutations on the stability N6AMT1-TRMT112 complex with FoldX_4 software using the data of crystal structure (PDB: 6H1E). We found that four of the seven energetically most important amino acids for dimerization were absent in iso2 (Figure S1B). In addition to hydrophobic amino acids, Arg111 was also in the list of amino acids having a destabilizing effect and thus had an impact for heterodimerization. However, the stability value for Asp117 was 0.4 kcal/mol (values between 0.5 and –0.5 were considered neutral) suggesting that this mutation might not have an effect, and the inability of N6AMT1-R111D117/AA mutant to form a complex with TRMT112 was caused mainly by substitution of N6AMT1 Arg111 to alanine. The crystal structure of the N6AMT1-TRMT112 complex with amino acids contributing to the formation of heterodimerization surface are depicted in Supplementary Figure S1C. TRMT112 Glu51 as a potential interacting partner of N6AMT1 Arg111 is also shown.

### 3.3. WBSCR22 and N6AMT1 Proteins Are Differently Regulated by Down-Regulation of TRMT112

**Figure 5:**
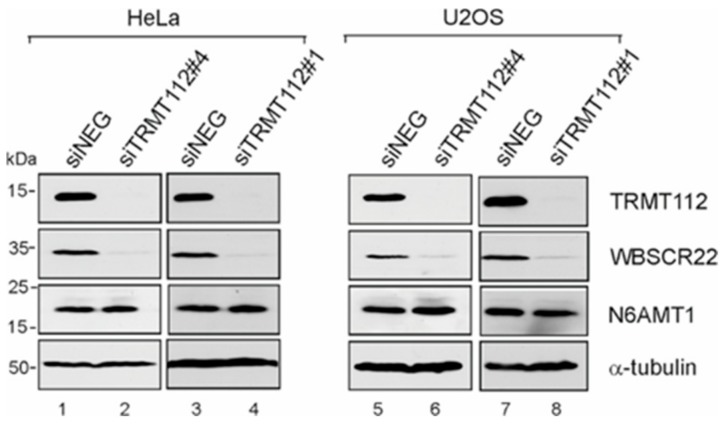
N6AMT1 and WBSCR22 respond differently to down-regulation of TRMT112. Protein expression in HeLa and U2OS cells transfected with siNeg and siTRMT112 was detected by western blot analysis with antibodies against TRMT112, WBSCR22, N6AMT1 and α-tubulin.

We and others have previously shown that the knock-down of TRMT112 with different TRMT112 siRNAs resulted in a significant decrease of WBSCR22 protein level in HeLa as well as U2OS cells [8,9]. To study the effect of TRMT112 down-regulation for the stability of the N6AMT1 protein, siRNAs that efficiently reduced the TRMT112 as well as WBSCR22 protein levels in our previous study were used [9]. Treatment of both HeLa and U2OS cells with siTRMT112 reduced the expression of TRMT112 and WBSCR22, but not N6AMT1 protein level in both cell lines tested (Figure 5). These results suggest that two TRMT112 interaction partners, WBSCR22 and N6AMT1, are differently regulated by TRMT112 in human cells, the stability of N6AMT1 is not influenced by down regulation of the TRMT112 protein.

## 4. Discussion

N6AMT1 is a DNA N6-adenine as well as protein methyltransferase that requires the TRMT112 protein for its activity in cells. The human genome encodes two different isoforms of N6AMT1, isoform 1 containing six exons and alternatively spliced isoform 2, where the fourth exon, encoding the substrate binding domain, is missing. Both isoforms are equally expressed on the mRNA level, but only the major isoform is detected on the protein level in human cells. We showed that the alternatively spliced isoform of N6AMT1, iso2, was expressed, but was not able to form a complex with the TRMT112 protein, and was rapidly degraded from the cells. This added an extra layer to the post-translational control mechanism to ensure that only the N6AMT1 major isoform was stably expressed on the protein level.

Two isoforms of N6AMT1, named HEMK2α and HEMKβ in human and PRED28α and PRED28β in mouse in previous studies, have shown to be expressed in all tissues in mice [25]. Figaro et al. has shown that the major isoform, but not the alternatively spliced isoform 2, missing the substrate binding domain, is functional in complementing the *Mtq2* deletion in yeast [10]. This suggests that only the major isoform of N6AMT1 is biologically active and is therefore preferentially expressed in the cells. Why this is important? First, only the N6AMT1-TRMT112 complex possesses protein, glutamine as well as lysine methyltransferase activities [20,23], so the isoform 2, that is not able to bind TRMT112, does not function as a protein methyltransferase. Second, structural studies in *Saccharomyces cerevisiae* have shown that the yeast Mtq2-Trm112 interaction triggers a conformational change of Mtq2, which strongly increases the binding of AdoMet [23]. AdoMet binding is a prerequisite for the methyltransferase activity, interaction with TRMT112 increases the AdoMet binding and enzymatic activity of N6AMT1 [10,23]. Third, there is a theoretical possibility that the expression of isoform 2 is hindered, because it lacks the substrate binding domain and thus may interfere in the activity of the full-length protein as a competitive inhibitor.

The N6AMT1 protein is a methyltransferase with a protein glutamine methyltransferase as well as histone H4 lysine 12 methyltransferase and DNA N6-adenine methyltransferase activities. Disruption of the mouse *N6amt1* gene resulted in embryonic lethality [28], such a strong phenotype suggests that this protein has an important biological functions in mammalian cells. First, through its glutamine methyltransferase activity it regulates the termination of translation mediated by ETF1 and the biological activities of several other proteins including CHD5 and NUT [10,22]. Second, independently of ETF1 methylation, N6AMT1 monomethylates H4K12 and controls the proliferation of prostate cancer cells through the expression of genes involved in the cell cycle [20]. Third, N6AMT1 was recently described as an N6-adenine methyltransferase [21,29]. The dynamic changes in N6-methyladenine levels on DNA are associated with LINE transposons activity in mouse brain and embryonic stem cells, which suggests that N6-methyladenine has an important role in neuropsychiatric disorders and epigenetic regulation during early embryogenesis [30,31]. N6AMT1 mediated accumulation of N6-methyladenine is dynamically regulated in the mammalian genome. It is required for the extinction of conditioned fear and serves as an epigenetic signal for regulating the activity- and learning-induced gene expression [29]. The N6-methyladenine level has shown to be downregulated in human cancer tissues, the reduction of genomic N6-adenine methylation promotes tumorigenesis [21]. 

**Figure 6:**
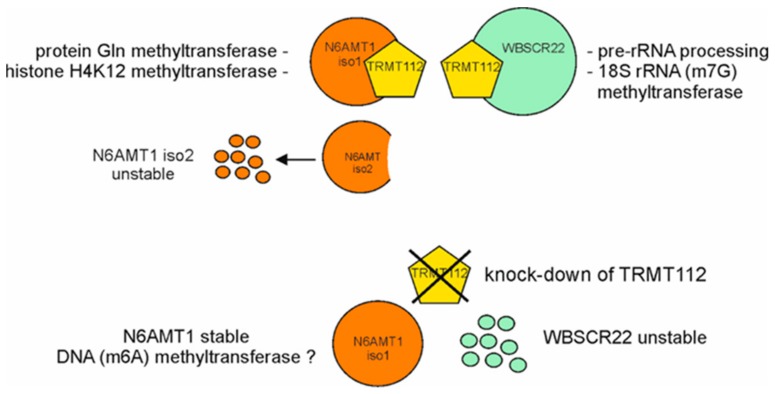
Model depicting the regulation of N6AMT1 and WBSCR22 proteins by co-factor TRMT112. N6AMT1 and WBSCR22 require the common co-factor TRMT112 for their stability and activity. The human genome encodes two different isoforms of N6AMT1, the major isoform (iso1) and the alternatively spliced isoform (iso2), where the substrate binding motif is missing. N6AMT1iso2 is not able to interact with TRMT112 and when translated, is rapidly degraded from the cells. Knock-down of TRMT112 results in degradation of WBSCR22, but does not affect the stability of N6AMT1. The known biological activities of N6AMT1 and WBSCR22 proteins (references in the text) are shown.

N6AMT1 and WBSCR22 are differently regulated by their mutual interaction partner TRMT112. We have previously shown that down-regulation of TRMT112 with siRNAs caused a significant decrease in WBSCR22 protein levels in human cells [9]. Here we showed that the siTRMT112 treatment reduced the expression of TRMT112 and WBSCR22, but not N6AMT1 protein levels in two cell lines, U2OS and HeLa cells. This suggests that WBSCR22 and N6AMT1 proteins were differently regulated by their cofactor TRMT112 in human cells. These findings differed from the results shown in yeast, where the knock-down of TRMT112 reduced the levels of both Bud23 and Mtq2, showing that Trm112 protein was required for the stability of both proteins [32]. In mammalian cells, association with TRMT112 is required for the stability of WBSCR22 and for the protein methyltransferase activity of N6AMT1 (Figure 6). Interestingly, N6AMT1 alone was sufficient to methylate the adenine in DNA in vitro [21], raising the possibility that N6AMT1 protein alone and in complex with TRMT112 recognize different substrates and possess either DNA or protein methyltransferase activities, respectively. This may explain why N6AMT1 is also stable without TRMT112 and is not degraded from the cells. Figaro et al. showed that N6AMT1 (HEMK2) can also be purified alone, but does not have glutamate methyltransferase activity of ETF1, adding separately purified TRMT112 was sufficient to restore the methylation activity in vitro [10]. In their work, the methylation efficiency was proportional to the amount of TRMT112 added, suggesting that the N6AMT1-TRMT112 complex is formed post-translationally and is dynamic.

The crystal structures of three eukaryotic Trm112-MTase complexes show that Trmt112 interacts in a very similar way with its MTase partners Mtq2, Bud23 and Trm9 and that the hydrophobic interactions are the major force driving heterodimer formation [23]. In the current study, we analyzed the heterodimerization surface of human N6AMT1-TRMT112 complex and found that four amino acids of seven, including two hydrophobic (N6AMT1 Leu108 and Leu112), which are involved in the formation of dimerization surface, were absent in iso2 (Figure S1B). In addition to hydrophobic amino acids, N6AMT1Arg111 had also a considerable impact on the formation of the N6AMT1-TRMT112 complex. According to the crystal data, N6AMT1Arg111 is able to form a salt bridge with Glu51 of TRMT112 and at least four hydrogen bonds with Glu51, Asn8 and Lys49 of TRMT112. Very recently the crystal structure of mammalian specific methyltransferase METTL5 in complex with TRMT112 was solved [33]. This allows us to compare the interaction surfaces of different methyltransferases with the common co-factor TRMT112 in the future.

Alternative splicing of mRNA produces a wide variety of differently spliced RNA transcripts that may be translated into diverse protein products. This is commonly believed to be a major source of cellular protein diversity. However, large-scale proteomics experiments identify few alternative isoforms suggesting that the vast majority of genes have a single dominant splice isoform leading to speculations that alternative transcripts may not even be translated into proteins [34]. In this study, we showed that both N6AMT1 isoforms were translated into proteins, but only the major isoform was stable in human cells. The human genome encodes approximately two hundred methyltransferases, only some of them have been shown to have alternative isoforms, which add a layer of complexity to the functional roles they play in normal and disease contexts [5,35]. For instance, protein arginine methyltransferase PRMT1 encodes eight different isoforms that can differ by their catalytic activity as well as substrate specificity and it is assumed that catalytically inactive isoform lacking dimerization arm could act as a competitive inhibitor for PRMT1 in cancer cells by shielding substrates from access to active isoform oligomers [36,37]. DNA methyltransferase DNMT3B is expressed in more than 30 isoforms, whereas several catalytically active and inactive isoforms act as accessory proteins in coordinating DNA methylation [38,39,40]. In contrast, N6AMT1 methyltransferase has only one major isoform that is expressed as a stable protein, the level of the alternatively spliced isoform is strictly controlled. Alternative splicing is a powerful means of enhancing protein diversity. It is estimated that over 60% of human genes are alternatively spliced, the challenge is to find out how much of them are actually translated as stable proteins and have biological functions.

## 5. Conclusions

TRMT112 is a small protein that acts as a cofactor for different methyltransferases involved in rRNA, tRNA, DNA and protein methylation. The TRMT112 protein interacts with at least four methyltransferases, including N6AMT1 and WBSCR22, in mammalian cells and is required for the activity and stability of these enzymes. Here we show that interaction with TRMT112 was also required for the regulation of the expression of N6AMT1 isoforms in cells, suggesting that TRMT112 was involved in cellular quality control ensuring that the isoform with missing substrate binding domain was eliminated from the cells. In addition, N6AMT1 and WBSCR22 were differently regulated by their mutual partner TRMT112, down-regulation of TRMT112 reduced the expression of WBSCR22, but not N6AMT1 levels in the cells. Our data suggest that TRMT112 was involved in many different cellular regulatory pathways and the network was more complex than initially expected.

## Supplementary Materials

The following are available online at https://www.mdpi.com/2218-273X/9/9/422/s1, Figure S1: Comparison of the crystal structure of N6AMT1-TRMT112 complex (PDB: 6H1E) with the model used in the current study.
