# Supplementary material for: The Common Partner of Several Methyltransferases TRMT112 Regulates the Expression of N6AMT1 Isoforms in Mammalian Cells
Source: Biomolecules. 2019 Aug 28;9(9):422. doi: 10.3390/biom9090422 (PMC6769652; doi:10.3390/biom9090422)
Supplement: Supplementary file 1 [file biomolecules-09-00422-s001.pdf]

A

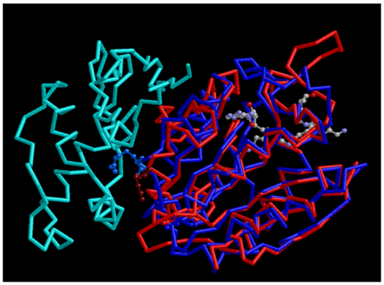

B

Amino acid	A6NMT1 Position	complex stability ddG (kcal/mol)
**Gly**	**107**	2,42
Val	100	2,35
**Arg**	**111**	2,27
Ile	101	1,99
Leu	72	1,80
**Leu**	**108**	1,34
**Leu**	**112**	1,07

C

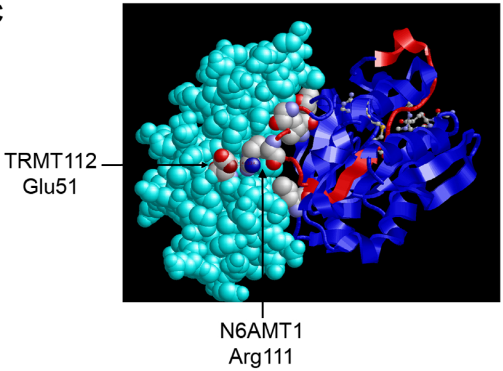

Figure S1. (A) Comparison of the crystal structure of N6AMT1-TRMT112 complex (PDB: 6H1E) with the model used in the current paper. For N6AMT1, the model created in the current study is coloured red and crystal structure blue, and TRMT112 is shown in cyan. N6AMT1 Arg111 is marked with blue (crystal) and red (model), SAH (S-adenosyl-L-homocysteine and peptide with CPK-coloured "Ball and Stick", respectively.
(B) Calculated energetic effect of alanine mutation on the stability of N6AMT1-TRMT112 complex based on crystal structure 6H1E. The FoldX_4 software [1] was used to evaluate the effect of Ala mutagenesis to complex stability. First, the protein sidechain conformation in PDB entry 6H1E was energy optimised with running ´RepairPDB´ command (temperature parameter set to 298K and ionStrength parameter to 0.15.), then the same was applied to coordinates of 6H1E where atoms of TRMT112 were removed. ´Ala Scan´ command was applied to energy minimized structures and specific effect to complex formation (ddG(complex)-ddG(monomer)) was calculated for each position. Most destabilizing ddG values of amino acids on the heterodimerization surface are shown. Amino acids lacking in iso2 are shown bold. The complex stability value for N6AMT1 Asp117 was 0.4 kcal/mol.
(C) The crystal structure of N6AMT1-TRMT112 complex. N6AMT1 is shown as a blue ribbon structure and TRMT112 cyano-colored spacefill. Amino acids on the dimerization surface, Arg111, Val100, Ile101, Gly107 and Leu72 from N6AMT1 and Glu51 of TRMT112 are shown with CPK-colored spacefill. Amino acids present in isoform 1, but missing in isoform 2 are colored red.

[1] Schymkowitz, J.; Borg, J.; Stricher, F.; Nys, R.; Rousseau, F.; Serrano, L. The FoldX web server: an online force field. *Nucleic Acids Res*. **2005**, 33, W382-388.
